# Compact Double-Layer FR4-Based Focusing Lens Using High-Efficiency Huygens’ Metasurface Unit Cells

**DOI:** 10.3390/s20216142

**Published:** 2020-10-28

**Authors:** Kd M. Raziul Islam, Sangjo Choi

**Affiliations:** Department of Electrical Engineering, University of Ulsan, Ulsan 44610, Korea; 20195153@mail.ulsan.ac.kr

**Keywords:** metasurface, focusing lens, Huygens’ principle, surface electric and magnetic currents

## Abstract

High transmission efficiency metasurface unit cells have been designed based on surface electric and magnetic impedances derived from Huygens’ principle. However, unit cells for low transmission loss (<1 dB) over a wide transmission phase range require at least three metallic layers, which complicates the unit cell design process. In this paper, we introduce high-efficiency Huygens’ metasurface unit cell topologies in double-layer FR4 printed circuit board (PCB) by implementing surface electric and magnetic current using the top and bottom metallic patterns and via drills. Eleven unit cells were optimized for wide phase coverage (−150° to 150°) with a low average transmission loss of −0.82 dB at 10 GHz. To demonstrate the high-efficiency of the designed unit cells, we designed and fabricated two focusing lenses with dimensions of near 150 × 150 mm (5λ × 5λ) to focus a spherical beam radiated from short focal distances (f = 100 and 60 mm). The fabricated focusing lens showed 12.87 and 13.58 dB focusing gain for f = 100 and 60 mm at 10 GHz, respectively, with a 1 dB fractional gain bandwidth of near 10%. We expect that the proposed focusing lens based on high-efficiency double-layer metasurface unit cells can help realize compact and high-gain focusing lens-integrated antenna systems.

## 1. Introduction

Metasurface is a two-dimensional structure with subwavelength particles or unit cells for manipulating propagation direction [1,2,3,4,5], polarization [6,7,8,9,10,11], and orbital angular momentum (OAM) [12,13,14,15] of electromagnetic (EM) waves. The ground-breaking feature from the metasurface was introduced from extensive review papers [16,17,18,19,20], and metasurface-based lenses [21,22,23], antennas [24,25,26,27], and holographic imaging [28,29] have been actively studied. After the advent of a popular metasurface design using the generalized Snell’s law [1], which deals with a phase gradient (scalar) on the surface, Huygens’ principle was utilized to improve the efficiency of wave-refracting metasurfaces by introducing surface electric and magnetic currents (vector quantities) [2]. Using Huygens’ principle, a metasurface was modeled as a two-dimensional boundary and the required electric and magnetic surface impedances to control the propagation direction of a transmitted wave were achieved [2]. Metasurface design based on Huygens’ principle is theoretically sound; however, it is not straightforward to implement electric and magnetic surface impedances that cover a wide transmission phase range with high transmission efficiency in a planar 2D structure, e.g., printed circuit board (PCB). In [2], Pfeiffer et al. designed electric and magnetic dipoles in two-layer PCB strips, but had to stack the strips between air space to align the vector components of the incident electric and magnetic fields with the corresponding surface impedance vectors. More fabrication-friendly unit cells with the required surface impedances have been designed and laid out in two-layer PCBs, but the unit cells were not able to maintain low transmission loss (<1 dB) for all the required surface impedances, especially high electric and magnetic resonance cases [30,31,32,33,34,35,36,37]. For better efficiency, a three-layer PCB capable of generating a loop current from the first and third layers using two vias was implemented [38,39,40]. Recently, in the same three-layer PCBs, symmetric and asymmetric unit cells with two magnetic dipoles (dogbone shape) on the top and bottom layer and an electric dipole (capacitor-loaded dipole) in the middle layer have been implemented [25,26,41,42]. Despite the higher transmission efficiency of these three-layer unit cells, the drawbacks can be increased geometrical parameters that need to be tuned and interference by waves propagating along the multiple layers, which require optimization.

In this paper, we introduce double-layer metasurface unit cells with high transmission efficiency and wide phase coverage at 10 GHz by constructing electric and magnetic dipole structures based on Huygens’ principle. In the unit cells, electric and magnetic dipoles were implemented in 1.6 mm-thick (≈λ/18.8) double-layer FR4 PCB and via drills were used to form antisymmetric conducting loops for magnetic dipole resonance by connecting top and bottom metallic traces. Vias were also implemented in the electric dipole structure and the desired capacitance of the unit cell was achieved. Overall, we designed 11 unit cells to cover transmission phases from −150° to 150° and achieved a low average transmission loss of −0.82 dB at 10 GHz. To assess the practical performance of the unit cells, we designed two compact lenses with near 5λ × 5λ (152.1 × 156 mm) size to focus a spherical beam to specific focal points of 100 mm (f/D = 0.65) and 60 mm (f/D = 0.39). The lenses were designed for such low f/D values to be used for a compact and low profile transmitarray [43,44]. From the fabricated devices, radiated power from the half-wave dipole located at the focal distances was enhanced by 12.87 and 13.58 dB at 10 GHz for f/D = 0.65 and 0.39 cases, respectively, and a wide 1 dB gain bandwidth near 10% was demonstrated.

## 2. Huygens’ Metasurface Unit Cell Design

### 2.1. Unit Cell Design Method

The generalized sheet transition condition (GSTC) models a metasurface as electric and magnetic polarizability densities ($\alpha_{\mathrm{ES}}$ and $\alpha_{\mathrm{MS}}$) and relates those to the transmission coefficient (T) and reflection coefficients (R) of the metasurface [45]. Then, using Equations (1) and (2), $\alpha_{\mathrm{ES}}$ and $\alpha_{\mathrm{MS}}$ can be converted to the electric surface admittance ($Y_{\mathrm{es}}$) and magnetic surface impedance (Z_ms_), which are familiar concepts from Huygens’ principle. Here, we assume isotropic surface electric admittance and magnetic impedance.

(1) $$Y_{\mathrm{es}}={j\omega \varepsilon \alpha }_{\mathrm{ES}}$$

(2) $$Z_{\mathrm{es}}={j\omega \mu \alpha }_{\mathrm{MS}}$$

By the simple conversion, the normalized values of $Y_{\mathrm{es}}\eta_{0}$_0_ and $Z_{\mathrm{ms}}/\eta_{0}$ are given in terms of T and R in Equations (3) and (4). It is important to note that these simple equations can be derived when the propagation directions of the incident and transmitted waves are normal to the boundary [45]. More general equations with the arbitrary incident and transmitted angles were also derived in [46].

(3) $$Y_{\mathrm{es}}\eta_{0}=2\frac{1-T-R}{\;1+T+R}$$

(4) $$Z_{\mathrm{ms}}/\eta_{0}=2\frac{1-T+R}{1+T-R}$$

**Figure 1:**
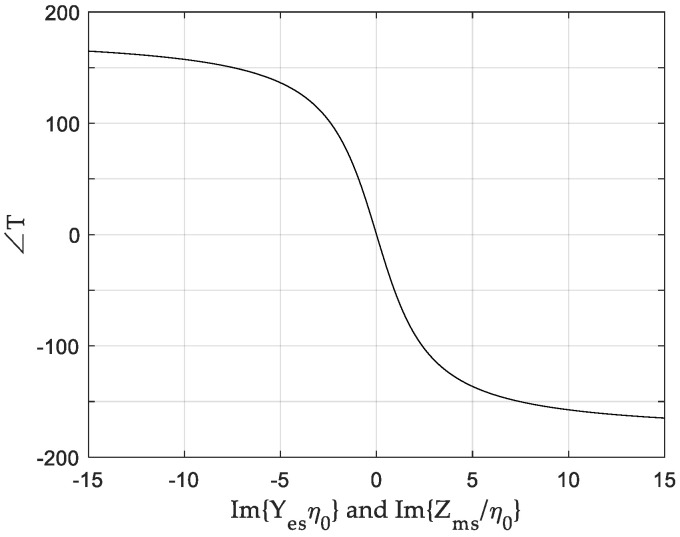
Transmission phases versus the imaginary part of the normalized surface electric admittance $I_{m}${Y_es_$\eta_{0}$} and the normalized surface magnetic impedance Im{$Z_{\mathrm{ms}}/\eta_{0}$}.

Here, $\eta_{0}$ is the intrinsic impedance of free space. Equations (3) and (4) indicate that a reflectionless (R = 0) metasurface requires the same values of $Y_{\mathrm{es}}\eta_{0}$ and $Z_{\mathrm{ms}}/\eta_{0}$. To maintain almost perfect transmission (T = 1) with wide transmission phase variation, only the imaginary part of $Y_{\mathrm{es}}$ and $Z_{\mathrm{ms}}$ should be utilized. Figure 1 shows that the simultaneous change of Im{$Y_{\mathrm{es}}\eta_{0}$} and Im{$Z_{\mathrm{ms}}/\eta_{0}$} from −15 to 15 covers transmission phases from −165° to 165°. Here, $Y_{\mathrm{es}}\eta_{0}$ and $Z_{\mathrm{ms}}/\eta_{0}$ are purely imaginary numbers.

### 2.2. Unit Cell Topology Design and Analysis

**Figure 2:**
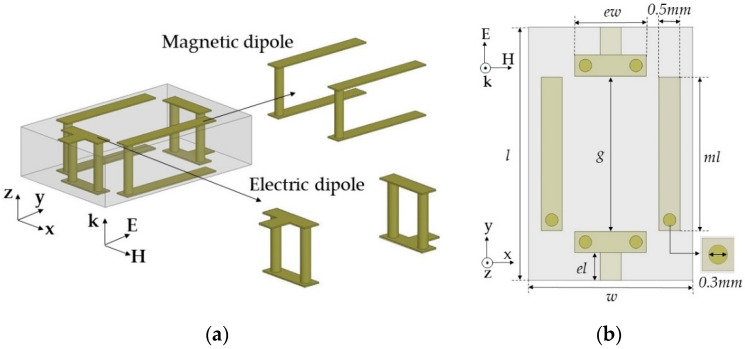
Electric and magnetic dipole in topology 1 from the (**a**) side–side view and (**b**) top view.

We designed unit cells to provide desired normalized $Y_{\mathrm{es}}$ and $Z_{\mathrm{ms}}$ values to cover a wide range of transmission phases (−150° ~ 150°) with low transmission loss (<1 dB) at 10 GHz. Instead of using one topology for all the phases, we designed three optimum unit cell topologies for specific transmission phase ranges. First, we designed a unit cell with a high positive $Y_{\mathrm{es}}$ and $Z_{\mathrm{ms}}$ to realize the −150° transmission phase with low transmission loss. Generally, electric dipole resonance can be implemented easily with capacitor-like structures and is stronger than magnetic resonance. To increase magnetic resonance strength, which should match the electric resonance strength, we implemented two magnetic dipoles on the sides and one electric dipole at the center using vias, as shown in Figure 2.

**Figure 3:**
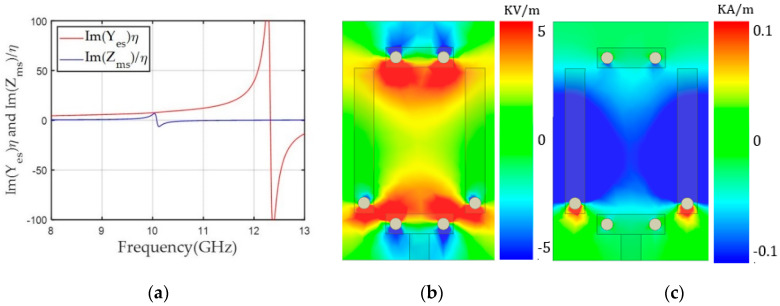
(**a**) Y_es_ and Z_ms_ values of the unit cell according to frequency for a transmission phase of -150°. (**b**) The y component of the electric field (E_y_). (**c**) The x component of the magnetic field (H_x_) along the x-y plane in the middle of the unit cell at 10 GHz.

Here, the alignment of both dipoles was based on a y-polarized TEM wave propagating along the z-axis. The unit cells were simulated using Ansys high frequency structure simulator (HFSS) and the excitation and boundary conditions were used with wave ports and perfect electric conductor (PEC)/perfect magnetic conductor (PMC) boundaries, respectively. In the design, we used a 1.6 mm-thick double-layer FR4 substrate with a relative permittivity (ε_r_) of 4.3 and loss tangent (tan δ) of 0.008. The unit cell area was fixed to a length (l) of 6 mm and width (w) of 3.9 mm. We used the rectangular-shaped unit cell to support long asymmetric current flow and a close space between the two magnetic dipoles to achieve a high Z_ms_ value and a low discretization error along the x-axis. Geometrical parameters of the unit cell included ml (the length for the magnetic dipole), g (the gap between the capacitively coupled electric dipoles), and ew (the width of the capacitor of the electric dipoles). Last, el indicates the electric dipole length, which is dependent on g. Therefore, we varied ml for the desired $Y_{\mathrm{es}}$ values and g and ew for the $Y_{\mathrm{es}}$ tuning. One benefit of this unit cell topology is that electric and magnetic resonance can be controlled independently because both electric and magnetic structures are implemented perpendicularly by the intuition of Huygens’ surface electric and magnetic currents. Finally, a unit cell with ml = 3.64 mm, g = 3.68 mm, and ew = 1.7 mm achieved high $Y_{\mathrm{es}}$ and $Z_{\mathrm{ms}}$ values of 7.55 and 5.87, respectively, with an S_21_ of −0.96 dB and −150° transmission phase. To balance $Y_{\mathrm{es}}$ and $Z_{\mathrm{ms}}$, $Y_{\mathrm{es}}$ resonance where the peak of Y_es_ occurs has to be located near 12.4 GHz, which was implemented with lower capacitance by using a wide gap distance (g = 3.68 mm) between the two electric dipoles, as shown in Figure 3b. However, the $Z_{\mathrm{ms}}$ resonance should be near 10 GHz to achieve a sufficiently high $Z_{\mathrm{ms}}$ value to match the $Y_{\mathrm{es}}$ value at 10 GHz. Figure 3c shows a high magnetic field excited along the x-axis due to current flows on the magnetic dipoles.

**Figure 4:**
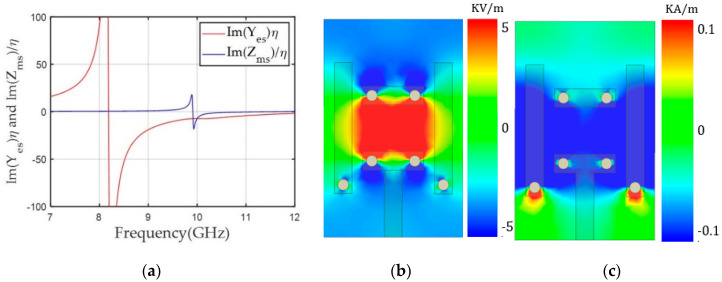
(**a**) Y_es_ and Z_ms_ values of the unit cell along frequency for a transmission phase of +150°. (**b**) The y component of the electric field (E_y_). (**c**) The x component of the magnetic field (H_x_) along the x-y plane in the middle of the unit cell at 10 GHz.

Next, we reduced the $Y_{\mathrm{es}}$ and $Z_{\mathrm{ms}}$ resonance frequencies to achieve balanced negative values for both $Y_{\mathrm{es}}$ and $Z_{\mathrm{ms}}$ at 10 GHz for the +150° transmission phase, as shown in Figure 4a. Based on the unit cell with a transmission phase of –150°, the gap (g) of the electric dipole was lowered up to 1.30 mm and the magnetic dipole length (ml) was decreased to 3.61 mm. This structure moved the Y_es_ and $Z_{\mathrm{ms}}$ resonance frequencies to 8.2 and 9.8 GHz, respectively, and achieved balanced $Y_{\mathrm{es}}$ (−7.19) and $Z_{\mathrm{ms}}$ (−7.15) at 10 GHz, providing −1.1 dB S_21_ with +150° phase. Figure 4b,c shows the unit cell structure for the +150° phase and E_y_ and H_x_ on the x–y plane in the middle of the structure. E_y_ was confined near the gap of the electric dipole’s capacitor structure, which was responsible for the lower resonance frequency of $Y_{\mathrm{es}}$ compared to the unit cell for the −150° phase. Figure 4c shows that the strong x components of the magnetic field were distributed in the middle of the magnetic dipoles, lowering the resonance frequency of $Z_{\mathrm{ms}}$ and yielding a negative $Z_{\mathrm{ms}}$ value at 10 GHz.

**Figure 5:**
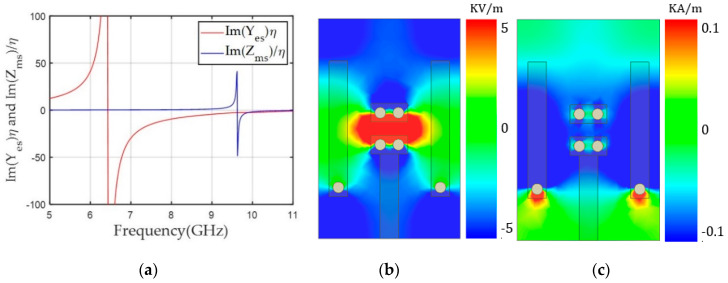
(**a**) Y_es_ and Z_ms_ values of the unit cell according to frequency for a transmission phase of +120°. (**b**) The y component of the electric field (E_y_). (**c**) The x component of the magnetic field (H_x_) along the x-y plane in the middle of the unit cell at 10 GHz.

We realized the unit cells for the other positive phases from +120° to 0° by lowering negative $Y_{\mathrm{es}}$ and Z_ms_ values at 10 GHz. To do that, we lowered the resonance frequency of $Y_{\mathrm{es}}$ and $Z_{\mathrm{ms}}$ by reducing the gap of the electric dipole (g) and tuning the electric dipole width (ew), and increasing the length of the magnetic dipole (ml), respectively. Figure 5a shows the normalized $Y_{\mathrm{es}}$ and $Z_{\mathrm{ms}}$ values from the unit cell for +120° with g = 0.37 mm, ew = 1.0 mm, and ml = 3.70 mm. The balanced $Y_{\mathrm{es}}$ and $Z_{\mathrm{ms}}$ values of −3.5 and −3.3 at 10 GHz are shown; this condition provided a low S_21_ of −1.1 dB. As shown in Figure 5b,c, E_y_ and H_x_ from the unit cell with +120° had a similar confined E_y_ field near a smaller gap of the electric dipole and stronger magnetic field due to a longer ml compared to the unit cell for the +150° phase. This phenomenon corresponded to lower $Y_{\mathrm{es}}$ and $Z_{\mathrm{ms}}$ resonance frequencies in the +120° phase structure. We decreased the $Y_{\mathrm{es}}$ and $Z_{\mathrm{ms}}$ resonance frequencies by using a more confined E_y_ and higher H_x_, and finally covered phases from +90° to 0°. Detailed geometries and $Y_{\mathrm{es}}$ and $Z_{\mathrm{ms}}$ values are shown in Table 1.

**Figure 6:**
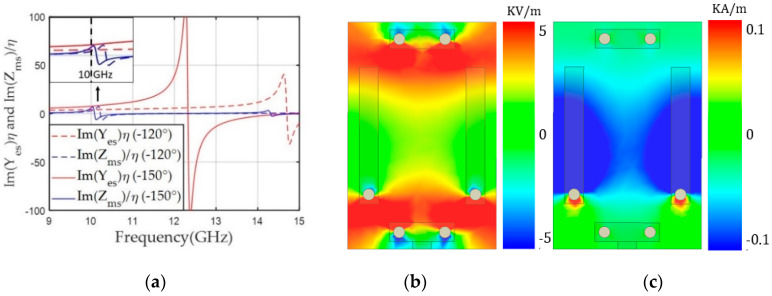
(**a**) $Y_{\mathrm{es}}$ and $Z_{\mathrm{ms}}$ values of the unit cell according to frequency for transmission phases of −150° and −120°. (**b**) The y component of the electric field (E_y_) for the transmission phase of −120°. (**c**) The x component of the magnetic field (H_x_) for −120° along the x-y plane in the middle of the unit cell at 10 GHz.

For negative transmission phases, we used the unit cell for −150° and achieved −120° by increasing the $Y_{\mathrm{es}}$ and $Z_{\mathrm{ms}}$ resonance frequencies. For lower effective capacitance and inductance of the unit cell, the gap (g) between the electric dipole was increased from 3.68 to 4.60 mm and the magnetic dipole length (ml) was shortened from 3.64 to 3.61 mm. Finally, lower positive $Y_{\mathrm{es}}$ (3.9) and $Z_{\mathrm{ms}}$ (2.8) values at 10 GHz were achieved with a low S_21_ of −0.72 dB. Figure 6a shows the $Y_{\mathrm{es}}$ and $Z_{\mathrm{ms}}$ values according to frequency for the unit cells for the −150° and −120° phases. Lower Y_es_ and $Z_{\mathrm{ms}}$ values were observed for the −120° case at 10 GHz due to the higher Y_es_ resonance frequency near 14.8 GHz. The lower $Z_{\mathrm{ms}}$ value in the −120° case was due to a resonance frequency shift to a higher frequency near 10.2 GHz. Figure 6b shows that E_y_ for the unit cell with the −120° phase was distributed over a larger area than observed for the −150° case (Figure 3a). Figure 6c for H_x_ also shows lower magnetic fields between two magnetic dipoles. Both trends correspond to higher resonance frequencies for both $Y_{\mathrm{es}}$ and $Z_{\mathrm{ms}}$ and lower values at 10 GHz for the −120° case.

**Figure 7:**
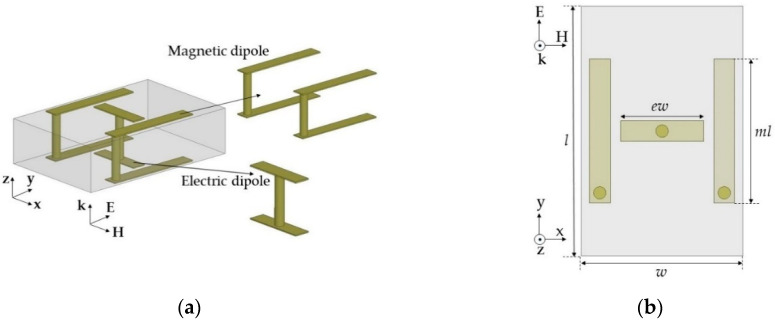

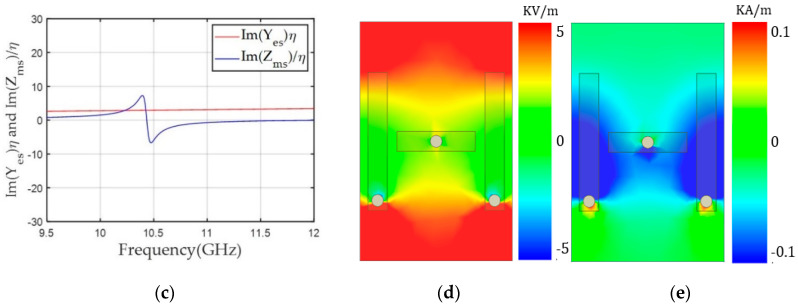
Electric and magnetic dipoles in the topology 2 structure from the (**a**) side-side view and (**b**) top view. (**c**) $Y_{\mathrm{es}}$ and $Z_{\mathrm{ms}}$ values of the unit cell according to frequency for a transmission phase of −90°. (**d**) The y component of the electric field (E_y_). (**e**) The x component of the magnetic field (H_x_) along the x-y plane in the middle of the unit cell at 10 GHz.

For the −90° transmission phase, lower positive $Y_{\mathrm{es}}$ and $Z_{\mathrm{ms}}$ are needed; however, the gap of the electric dipole (g) cannot be further increased due to fabrication limits. Therefore, we used one electric dipole structure to provide a lower $Y_{\mathrm{es}}$ at 10 GHz and two magnetic dipoles were maintained, as shown in Figure 7a. The geometrical parameters are shown in Figure 7b; an ew of 2 mm and ml of 3.47 mm provided a normalized $Y_{\mathrm{es}}$ of 2.8 and $Z_{\mathrm{ms}}$ of 1.4, resulting in the achievement of a −90° phase with a low S_21_ of −0.72 dB. Figure 7c shows that $Y_{\mathrm{es}}$ and Z_ms_ values became lower at 10 GHz due to $Z_{\mathrm{ms}}$ and $Y_{\mathrm{es}}$ resonance frequencies higher than 10 GHz. Figure 7d,e shows that E_y_ was distributed over a larger area and H_x_ was weaker in the −90° case than the −120° case.

**Figure 8:**
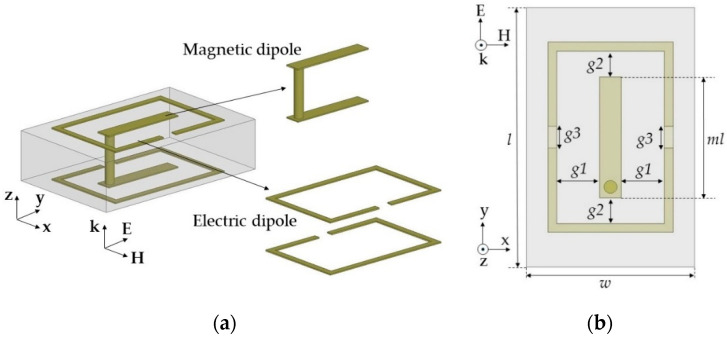
Electric and magnetic dipoles in the topology 3 structure from the (**a**) side–side view and (**b**) top view.

**Figure 9:**
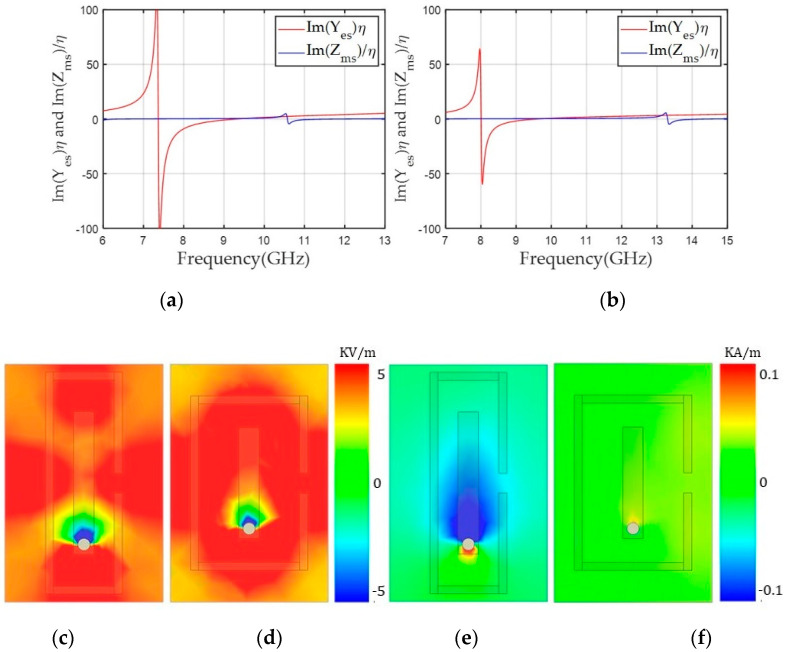
$Y_{\mathrm{es}}$ and $Z_{\mathrm{ms}}$ values of the unit cell according to frequency for transmission phases of (**a**) −60° and (**b**) −30°. The y component of the electric field (E_y_) for transmission phases of (**c**) −60° and (**d**) −30°. The x component of the magnetic field (H_x_) for transmission phases of (**e**) −60° and (**f**) −30° along the x-y plane in the middle of the unit cell at 10 GHz.

To further reduce $Y_{\mathrm{es}}$ and $Z_{\mathrm{ms}}$ to cover the −60° and −30° transmission phases, we used one magnetic dipole in the center of the unit cell and located the electric dipoles around the boundary of the unit cell on the top and bottom layers, as shown in Figure 8a. Figure 8b shows the geometrical parameters of topology 3. The length of g3 was fixed at 0.5 mm and horizontal gaps (g1 and g2) between the magnetic and the electric dipole structures, and magnetic dipole length (ml) were mainly tuned to control the near-zero $Y_{\mathrm{es}}$ and $Z_{\mathrm{ms}}$ accurately such that $Y_{\mathrm{es}}$ and $Z_{\mathrm{ms}}$ became 0.7 and 0.4 for the −30° case. Here, ml and g2 determine the vertical length of the electric dipole. Finally, we achieved almost zero transmission loss for the −30° and −60° cases (−0.34 and −0.59 dB, respectively). This topology achieved near-zero $Y_{\mathrm{es}}$ values at 10 GHz because maximum $Y_{\mathrm{es}}$ resonance frequencies were located under 8 GHz and the zero-crossing $Y_{\mathrm{es}}$ frequency was near 10 GHz, as shown in Figure 9a,b. Similar to the other negative phase cases, Z_ms_ resonance frequencies were higher than 10 GHz, resulting in near-zero $Z_{\mathrm{ms}}$ values at 10 GHz. Figure 9c,d shows that E_y_ was distributed along the entire area of the unit cells for transmission phases of −60° and −30°, in contrast to the other negative phase cases. We attributed the wide distribution of E_y_ to the near-zero $Y_{\mathrm{es}}$ at 10 GHz for both cases. Compared to the −60° case, the −30° case showed a more distributed E_y_; therefore a slightly higher $Y_{\mathrm{es}}$ value was achieved in the −60° case. Figure 9e,f shows that the −60° case had higher magnetic fields along the x-axis, resulting in a higher $Z_{\mathrm{ms}}$ value at 10 GHz.

**Figure 10:**
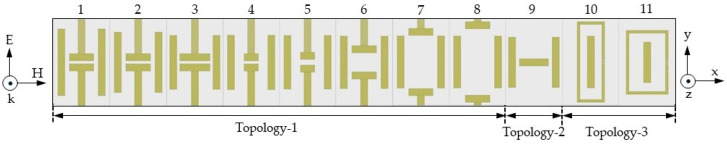
Top view of 11 unit cells with topologies 1, 2, or 3.

Figure 10 provides a top view of all the unit cells with topologies 1, 2, or 3. $Y_{\mathrm{es}}$ and $Z_{\mathrm{ms}}$ values of the corresponding unit cells and their transmission parameters are presented in Table 1. The highest and lowest transmission loss levels were −1.1 and −0.34 dB, respectively, and the average loss was −0.82 dB, which was lower than the average S_21_ of −1.04 dB reported for a similar double-layer PCB-based unit cell operating at 10 GHz [32]. Moreover, previously reported structures had a maximum transmission loss of −2.5 dB, while that of the proposed unit cells was −1.1 dB. These findings demonstrate that unit cell topologies optimized for specific transmission phases significantly reduce transmission loss of the overall unit cells.

### 2.3. Focusing Lens Design

**Figure 11:**
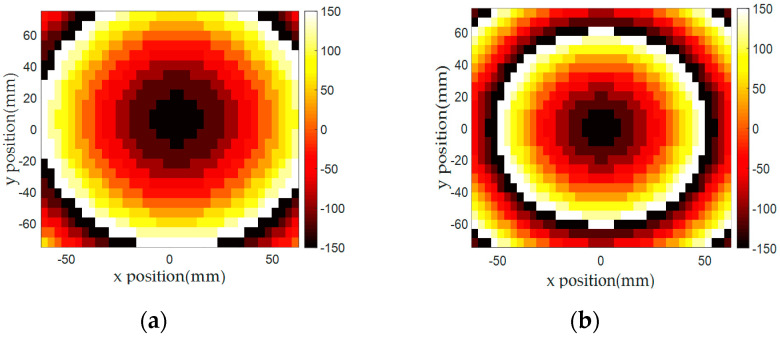

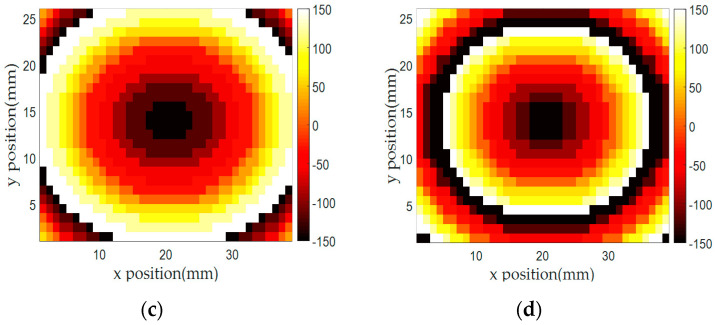
Required phase distributions calculated from Equation (5) of the focusing lens with (**a**) 100 mm and (**b**) 60 mm focal distances. Implemented phase distributions of the focusing lens with (**c**) 100 mm and (**d**) 60 mm focal distances.

Based on the high transmission efficiency of the 11 unit cells covering the −150° to 150° phases, we designed two focusing lens structures to collimate incident spherical waves radiated from short distances. Because the designed unit cells had subwavelength dimensions of w = 3.9 mm (λ/7.7) and l = 6 mm (λ/5), we were able to design a compact-sized (5λ × 5λ) array with short focal points (f) of 100 and 60 mm. Both 100 and 60 mm focal points means low f/D numbers of 0.65 and 0.39, respectively, that would be needed for a compact focusing lens-integrated system. The required transmission phases (ϕ_i_) to collimate the spherical waves from both focal points were determined from Equation (5), as shown in Figure 11a,b, and the corresponding unit cells were arranged accordingly. The target phases, drawn in Figure 11a,b, were discretized using 30° steps.

(5) $$\phi_{i}=k_{0}\left(R_{i}-f\right)+\phi_{0}$$

In Equation (5), $R_{i}$ is the distance between the center of the unit cell element and the focal point and f is the focal distance. We used the wavenumber ($k_{0}$) from the 10 GHz frequency and set $\phi_{0}$ to −150° as the default phase at the center of the array where $R_{i}$ and f are equal. One thing to note is that due to the unit cells’ compact size, steeper phase changes required for short focal distances could be realized with a low discretization error. Finally, we arranged different numbers of rectangular-shaped (3.9 × 6 mm) unit cells along the x-axis (39 cells) and y-axis (26 cells) to realize a near 5λ × 5λ-sized (152.1 × 156 mm) focusing lens. Figure 11c,d shows the implemented phase distribution of both focusing lenses. Because the calculated phases near the center of the focusing lenses were located in the middle of the 30° phase gap of the unit cells, a slight phase difference between Figure 11a,b and Figure 11c,d near the center area was allowed in the design process.

**Figure 12:**
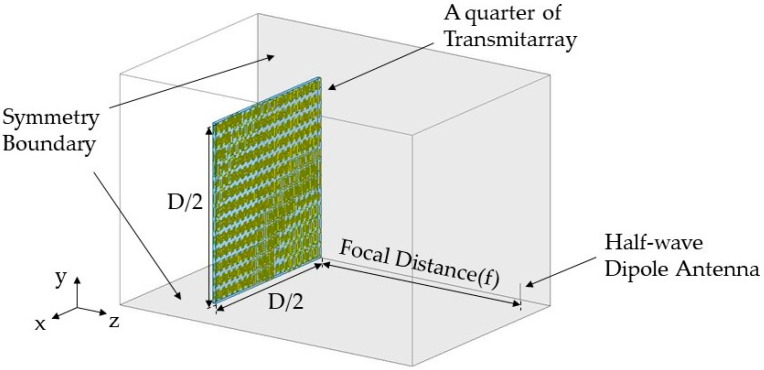
Simulation setup for the focusing lens with a half-wave dipole antenna as a feeding source in symmetry boundaries.

In simulations, both focusing lenses with 3D unit cell structures were implemented in HFSS and the focusing gain was calculated. For the simulation load reduction, a quarter of the focusing lens in the first quadrant was used with symmetric boundaries in the simulation tool (see Figure 12). A spherical wave as the incident wave was implemented by a half-wave dipole antenna operating at 10 GHz at a focal distance from the focusing lens. The focusing gain, defined as the gain from the focusing lens–combined dipole antenna minus the gain of the dipole antenna at the boresight [47,48,49] was calculated as 13.47 and 14.09 dB at 10 GHz for f = 100 and 60 mm, respectively. Gain patterns from the simulations were compared with those obtained experimentally (see below).

## 3. Fabrication and Measurement

**Figure 13:**
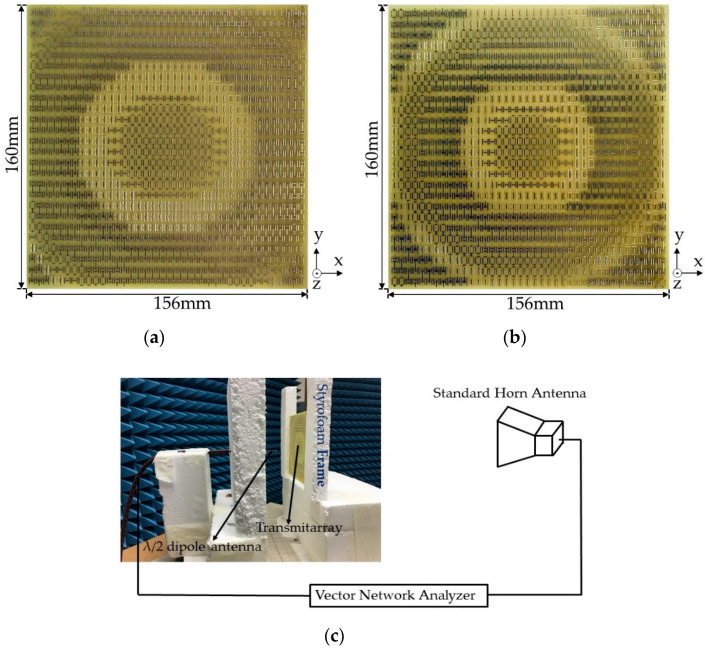
Top view of the fabricated focusing lenses with (**a**) 100 mm focal distance and (**b**) 60 mm focal distance. (**c**) Setup for measuring the focusing gain of the focusing lens using a half-wave dipole antenna as the feeding source and a standard horn antenna. Both antennas were connected to a vector network analyzer.

For experimental verification, both focusing lenses with the same PCB size (156 × 160 mm) for f = 100 and 60 mm were fabricated, as shown in Figure 13a,b. Extra spaces on the boundary were added for stable patterns and via fabrication based on the designed size of 152.1 × 156 mm. Figure 13c shows the measurement setup; a focusing lens was fed by a half-wave dipole operating at 10 GHz at a specified focal distance and the focused beam from the array was captured by a standard horn antenna. The transmission coefficient (S_21_) between the two antennas was measured using a vector network analyzer (Anritsu MS46122B, Anritsu Company, Morgan Hill, USA), and S_21_ values with and without the focusing lens were used to assess the focusing gain of the lens. For gain pattern measurements, the dipole antenna and the focusing lens were rotated horizontally in the same platform with a 1° step size and the measured pattern was compared with the simulation result. Distances between the dipole antenna and the focusing lens were maintained at the designed focal distances (100 and 60 mm).

**Figure 14:**
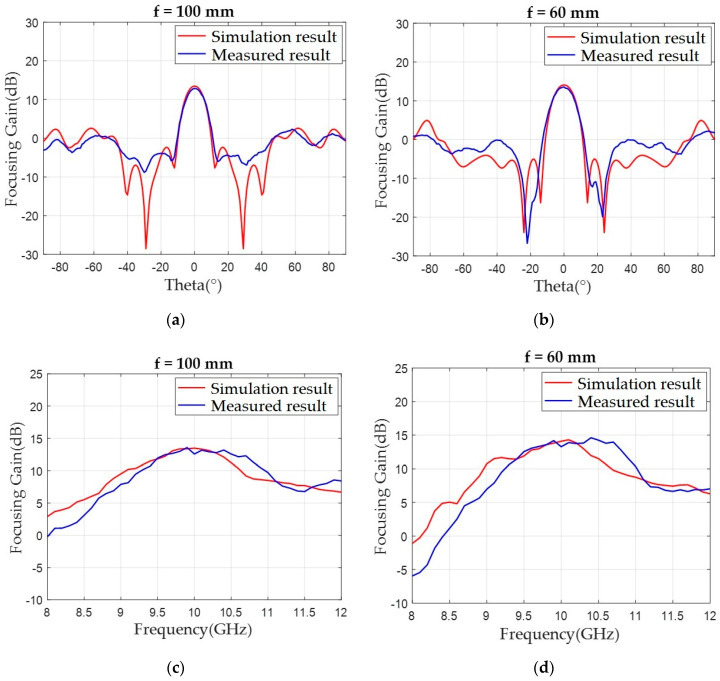
Simulated and measured and focusing gain patterns on the x-z plane with a function of theta at 10 GHz from the focusing lenses (**a**) with f = 100 mm and (**b**) with f = 60 mm. Simulated and measured focusing gain according to frequency from (**c**) f = 100 mm and (**d**) f = 60 mm.

Figure 14a,b demonstrates that the measured focusing gain at the boresight and sidelobe levels of both focusing lens correlated well with the simulation results at 10 GHz. The main beams at the boresight provided a focusing gain of 12.87 dB for f = 100 mm and 13.58 dB for f = 60 mm, which are approximately 0.5 ~ 0.7 dB lower than the simulated results of 13.47 and 14.09 dB, respectively. We attributed this difference to fabrication uncertainty, e.g., substrate property deviation in the focusing lens and the nonideal spherical wave radiation from the half-wave dipole antenna. However, a near 13 dB focusing gain level from a lossy FR4-based compact focusing lens (5.1λ × 5.2λ) with a low f/D value of 0.39 is noteworthy. In Figure 14a, the discrepancy between both data in terms of the dip of the main lobe is noticeable and the reason should be slightly perturbed current distributions on the fabricated lens due to the aforementioned manufacture-related factors. Focusing gain at the boresight according to frequency was also measured and compared with the simulation results, as shown in Figure 14c,d. The measured data showed a slightly broader bandwidth compared to the simulated one with a wide 1-dB gain fractional bandwidth of near 10%.

## 4. Discussion and Conclusions

We designed high transmission efficiency metasurface unit cells operating at 10 GHz in a double-layer FR4 with a thickness of 1.6 mm (λ/18.8) for wide transmission phase coverage. The unit cells’ physical structures were devised to implement perpendicularly directed surface electric and magnetic currents using the top and bottom metallic patterns and via drills. The proposed unit cells had three different topologies to control surface electric admittance (Y_es_) and surface magnetic impedance (Z_ms_) independently, providing compact (3.9 × 6.0 mm) 11 unit cells with an averaged −0.82 dB transmission loss and −150° ~ 150° transmission phases. Performance comparison with the referenced designs is shown in Table 2 and it is important to note that the designed unit cells achieved the lowest transmission loss even in a lossy FR4 (tan δ = 0.008) substrate compared to the recently reported double-layer unit cells from Table 2.

To assess the efficiency of the proposed unit cells experimentally, we designed two focusing lenses with a size of 156 × 160 mm (5.1λ × 5.2λ) to focus a radiated spherical beam in short focal distances (f = 100 and 60 mm). We expected the compact unit cell to maintain a low discretization error with low f/D numbers (f/D = 0.65 and 0.39), which required steep phase variation in the focusing lens design. The fabricated focusing lenses boosted the half-wave dipole’s gain more than 13 dB despite the short focal distances and achieved a wide 1-dB gain bandwidth of near 10%. Moreover, the focusing gain was increased by 0.7 dB with a focal distance change from 100 to 60 mm, demonstrating the low discretization error of the designed unit cells. From Table 2, the focusing gain levels of this work were lower within 3 dB compared to at least four times larger lenses in terms of center wavelength (λ), proving the higher focusing efficiency. Two slightly smaller lenses also showed 4 ~ 5 dB lower focusing gain values, and specifically, in [31], a compact (3.5λ × 3.5λ) and double-layer focusing lens with a low f/D of 0.29 showed focusing gain of 8.2 dB at 10 GHz. For a fair comparison, the aperture efficiencies using the measured focusing gain were calculated and a higher value of 6.88% from this work compared to 4.37% from [31] was shown. The higher efficiency from the designed lenses corresponds to the lower transmission loss from the proposed unit cells and manifests that higher spillover loss due to the broad radiation from the dipole antenna compared to the high gain patch antenna from [31] was also recovered. Moreover, the lens from [31] utilized narrow lateral dimensions for the phase coverage; it is thus subject to the narrow bandwidth (it was not reported). Finally, we expect that the high gain focusing lens with a low f/D based on the FR4-based high-efficiency double-layer unit cells can be utilized for low-profile beam-forming antenna systems in 5G and millimeter-wave communications.

## Figures and Tables

**Table 1:** Geometric parameters, simulated electric surface admittance ($Y_{\mathrm{es}}$), magnetic surface impedance ($Z_{\mathrm{ms}}$), transmission phases, and transmission loss of the 11 metasurface unit cells with topologies 1, 2, or 3.

Cell	Topology	g (mm)	g1 (mm)	g2 (mm)	ml (mm)	ew (mm)	Im (Y_es_)	Im (Z_ms_)	Trans. Phase (°)	Trans. Loss (dB)
1	1	0.20			4.60	1.7	0	0	0	−0.4
2	1	0.20			4.15	1.7	−1.1	−0.2	30	−1.1
3	1	0.20			3.72	2.1	−1.5	−0.9	60	−1.0
4	1	0.20			3.76	1.0	−2.4	−1.7	90	−1.0
5	1	0.37			3.70	1.0	−3.5	−3.3	120	−1.1
6	1	1.30			3.61	1.7	−7.19	−7.15	150	−1.1
7	1	3.68			3.64	1.7	7.55	5.87	−150	−0.96
8	1	4.60			3.61	1.7	3.9	2.8	−120	−0.72
9	2				3.47	2.0	2.8	1.4	−90	−0.72
10	3		0.5	0.8	3.6		1.4	0.88	−60	−0.59
11	3		1	0.6	2.8		0.7	0.4	−30	−0.34

**Table 2:** Performance comparison of the proposed focusing lenses with the referenced designs.

Ref.	Layer #	Freq. (GHz)	Loss Tangent	Max. Loss of Unit Cell (dB)	Lens Size (mm × mm)	Thickness (mm)	Bandwidth (3 dB/1 dB)	f/D	Focusing Gain (dB)
[35]	2	20	0.0014	−1.75	338 × 338 (22.5λ × 22.5λ)	1.575 (λ/9.5)	-/5.9	1.24	14.9 ^1^
[37]	2	28	0.0027	−1.63	165 × 165 (15.4λ × 15.4λ)	1.524 (λ/7.0)	13.3/-	0.95	16.4
[33]	2	26.2	0.001	−1.56	171.6 × 171.6 (15λ × 15λ)	1.5 (λ/7.6)	15.7/-	0.99	15.7 ^1^
[32]	2	13	0.0037	−2.5	328 × 328 (14.2λ × 14.2λ)	0.762 (λ/30)	3/-	0.8	11.5
[34]	2	10	0.005	−3	360 × 500 (12λ × 16.6λ)	2 (λ/15)	6/-	0.3	7.65
[50]	3	10.2	0.004	−2.75	376.5 × 376.5 (12.8λ × 12.8λ)	1.1 (λ/26.7)	9.8/-	0.8	14.4
[36]	2	6	-	−1.4	210 × 210 (4.2λ × 4.2λ)	2 (λ/25)	15/-	0.8	9
[31]	2	10	0.001	−1.4	104 × 104 (3.46λ × 3.46λ)	3 (λ/10)	-/-	0.29	8.2
This Work	2	10	0.008	−1.1	156 × 160 (5.1λ × 5.2λ)	1.6 (λ/18.8)	20/10	0.65/0.39	12.87/13.58

^1^ Focusing gain was calculated using the simulated gain from the feeding antenna.
